# Role of DEP domain-containing protein 1B (DEPDC1B) in epithelial ovarian cancer

**DOI:** 10.7150/jca.78423

**Published:** 2023-03-27

**Authors:** Yaxun Wu, Haibing Yin, Xingsong Zhang, Rong Shen, Xinghua Zhu, Meiqun Jia

**Affiliations:** 1 Department of Pathology, Affiliated Tumor Hospital of Nantong University, Nantong 226361, China; 2 Department of Gynecologic Oncology, Affiliated Tumor Hospital of Nantong University, Nantong 226361, China

**Keywords:** epithelial ovarian cancer, DEPDC1B, overall survival, progression-free survival, cell proliferation

## Abstract

Aberrant expression of DEPDC1B (DEP domain-containing protein 1B) has been shown to be associated with various types of malignant tumors. However, little is known about the role of DEPDC1B in epithelial ovarian cancer (EOC). The purpose of this study was to investigate the expression and role of DEPDC1B in EOC. Immunohistochemical staining of 60 high-grade serous ovarian cancer (HGSOC) showed that DEPDC1B expression was associated with response to first line chemotherapy, and DEPDC1B expression was higher in platinum-resistant patients than in platinum-sensitive patients. However, there was no association between DEPDC1B expression and age, preoperative CA125 level, ascites status, location, FIGO stage, and residual disease. Furthermore, our study demonstrated that increased DEPDC1B expression was correlated with reduced overall survival (OS) and progression-free survival (PFS) time in patients with HGSOC. Multivariate analysis showed that DEPDC1B independently predicted OS in patients with HGSOC. However, DEPDC1B expression was not an independent prognostic factor for PFS in patients with HGSOC. Moreover, our study demonstrated that DEPDC1B could promote the proliferation of OVCAR3 and SKOV3 cells by enhancing AKT phosphorylation at Ser473. Treatment with MK2206 and LY294002 significantly suppressed cell proliferation that is induced by DEPDC1B up-regulation in both OVCAR3 and SKOV3 cells. Together, these results revealed that DEPDC1B was an independent prognostic factor for OS in patients with HGSOC, and DEPDC1B may promote the proliferation of EOC cells via enhancing AKT phosphorylation at Ser473.

## Introduction

Epithelial ovarian cancer (EOC) is the first leading cause of gynecologic cancer deaths and ranks fifth in cancer deaths for women worldwide 1. Serous ovarian cancer is the most common histological subtype of EOC. About 70% of patients present with stage III and IV disease due to lack of effective screening programme 2. Despite recent advances in therapies, their prognosis remains dismal, and over 60% of patients diagnosed with late-stage EOC will recur or die within 5 years of their diagnosis 3, 4. Therefore, it is vitally important to elucidate the molecular mechanisms leading to EOC.

*DEPDC1B* (DEP domain-containing protein 1B) is localized on chromosome 5q12.1. As a signaling protein, DEPDC1B contains 2 conserved domains: a DEP domain and a RhoGAP domain 5. The DEP domain, which contains about 90 amino acids, acts as a scaffold to recruit signaling molecules to various subcellular locations 6. The RhoGAP domain is reported to play a vital role in Rho GTPase signaling 7. Currently, DEPDC1B is found to be overexpressed in some malignancies. Su et al. 5 reported that DEPDC1B was overexpressed in oral cancer, and upregulation of DEPDC1B could promote cell migration and invasion. Yang et al. 8 found that DEPDC1B might confer metastasis-related malignant phenotype to non-small cell lung cancer in a Wnt/β-catenin dependent manner. In addition, the expression of DEPDC1B was dramatically increased in prostate cancer tissues compared with normal prostate tissues. Increased DEPDC1B protein expression was found to be associated with advanced clinical stage, advanced T stage and lymph node metastasis. Furthermore, *DEPDC1B* mRNA was an independent prognostic factor for biochemical recurrence‑free survival in prostate cancer patients 9. Recently, Xu et al. 7 reported that the expression levels of DEPDC1B were significantly increased in malignant melanoma tissues compared with adjacent normal skin tissues. Knockdown of DEPDC1B could inhibit the proliferation of malignant melanoma cells, whereas knockdown of DEPDC1B could markedly promote cell apoptosis. However, the role of DEPDC1B in patients with EOC remains unknown. Given that DEPDC1B played a crucial role in the progression of a variety of human malignancies, we tested whether DEPDC1B played a promoting role in the development of EOC. The current study aimed to investigate the biological function and potential molecular mechanism of DEPDC1B in EOC.

## Materials and methods

### Patients and tissue samples

A total of 60 patients with high-grade serous ovarian cancer (HGSOC) were collected from the Department of Pathology, Affiliated Tumor Hospital of Nantong University from 2010 to 2020 and met the following criteria: (1) patients were histopathologically confirmed as HGSOC and treated with cytoreductive surgery and 6 cycles of platinum-based chemotherapy; (2) with complete follow-up, clinical and pathological information. The exclusion criteria were as follows: (1) with other serious diseases; (2) received other treatments, including preoperative chemotherapy, neoadjuvant chemotherapy, or immunotherapy. The median age of the patients was 53 years (range=39-76 years), and the median follow-up was 36 months (range=3-135 months). The study was approved by the Ethical Review Committee of the Affiliated Tumor Hospital of Nantong University.

### Immunohistochemistry and evaluation of immunohistochemistry

Automatic immunohistochemical staining was performed on a Dako Omnis autostainer (Dako, Agilent Technologies, Inc., Carpinteria, CA, USA). DEPDC1B antibody (1:100 dilution, bs-14278R, Bioss, Beijing, China) was incubated for 28 min. Detection was performed using the EnVision FLEX/ HRP reagent for 30 min. For immunohistochemical evaluation of DEPDC1B, the Histo-score (H-score) was calculated based on the staining intensity and percentage of stained cells as previously described 10. Briefly, the intensities were assessed using the following scores: 0, negative; 1, weak staining; 2, intermediate staining; 3, strong staining. The fraction of DEPDC1B-positive cells was scored 0-100. The H-score (range=0-300) was calculated by multiplying the intensity score and the fraction score. The patients were dichotomized into low (H-score <180) versus high (H-score ≥180) DEPDC1B expression using a cut-off threshold determined by X-tile software (Yale University).

### Cell culture, and lentivirus infection

The human normal ovarian cell line IOSE-80 was obtained from Shanghai Jingyuan Co., Ltd. (Shanghai, China). The EOC cell lines OVCAR3 and SKOV3, and the human hepatocellular carcinoma cell line BEL-7404 were obtained from Shanghai GeneChem Co., Ltd. (Shanghai, China). The human lung carcinoma cell line A549 was purchased from Cobioer Biosciences Company (Nanjing, China). IOSE-80 and BEL-7404 cells were routinely maintained in RPMI 1640 medium (GIBCO, Grand Island, NY, USA) supplemented with 10% fetal bovine serum (FBS, GIBCO). OVCAR3 cells were maintained in RPMI 1640 medium supplemented with 20% FBS. SKOV3 cells were maintained in McCoy's 5a medium (GIBCO) supplemented with 10% FBS. A549 cells were incubated in F12K medium supplemented with 10% FBS. Lentiviral DEPDC1B overexpression vector was purchased from the GeneChem. To establish cell lines stably expressing a high level of DEPDC1B, lentivirus-infected cells were selected with 2 μg/mL of puromycin (GeneChem).

### Antibodies, reagents, and western blot

Equal aliquots of total cellular protein were resolved by SDS-PAGE and transferred to PVDF membranes (Roche, Basel, Switzerland) for western blot using standard methods. Antibody concentrations and suppliers are as follows: DEPDC1B antibody (1:500 dilution, bs-14278R, Bioss), M2 Flag antibody (1:1000 dilution, Sigma-Aldrich, St Louis, MO, USA), Phospho-Akt (Ser473) antibody (1:1000 dilution, Cell Signaling Technology, Beverly, MA, USA), Akt antibody (1:1000 dilution, Cell Signaling Technology), c-Myc antibody (1:1500 dilution, Cell Signaling Technology), GAPDH antibody (1:5000 dilution, Proteintech Group, Wuhan, China), Peroxidase AffiniPure Goat Anti-Rabbit IgG (H + L) (1:10000 dilution, Jackson ImmunoResearch Laboratories, West Grove, PA, USA), Peroxidase AffiniPure Goat Anti-Mouse IgG (H + L) (1:10000 dilution, Jackson ImmunoResearch Laboratories). The PI3K inhibitor LY294002 and Akt inhibitor MK2206 were purchased from MedChemExpress (MCE, Shanghai, China).

### Colony formation assay

For colony formation analysis, the cells were digested with trypsin, counted and seeded onto 35-mm culture dishes at a density of 1×10^3^ cells per dish. The medium was changed every 3 days for 15 days. Colonies were fixed in 4% paraformaldehyde (Beyotime Biotech, Shanghai, China), and stained with crystal violet (Beyotime Biotech).

### EdU (5-ethynyl-2'-deoxyuridine) cell proliferation assay

Proliferating cells were evaluated by using the EdU assay kit (RiboBio, Guangzhou, China) according to the manufacturers' instructions. Briefly, cells were seeded onto 96-well plates, and incubated with EdU. After fixation with 4% paraformaldehyde and treated with 0.5% Triton X-100, cells were incubated with 1×Apollo reaction cocktail for 20 min. Subsequently, cell nuclei were stained with Hoechst reagent. Images were taken under an inverted microscope.

### Statistical analysis

The association between DEPDC1B expression and clinicopathological parameters was analyzed by Pearson's chi-square test or Fisher's exact test. Survival analysis was performed using the Kaplan-Meier method, and the log-rank test was used to compare survival curves. The Cox proportional hazards regression model was utilized for both univariate and multivariate analyses of survival. Comparison between groups was evaluated by Student's t-test. *P*-values <0.05 were considered statistically significant.

## Results

### DEPDC1B expression in HGSOC tissues

**Figure 1:**
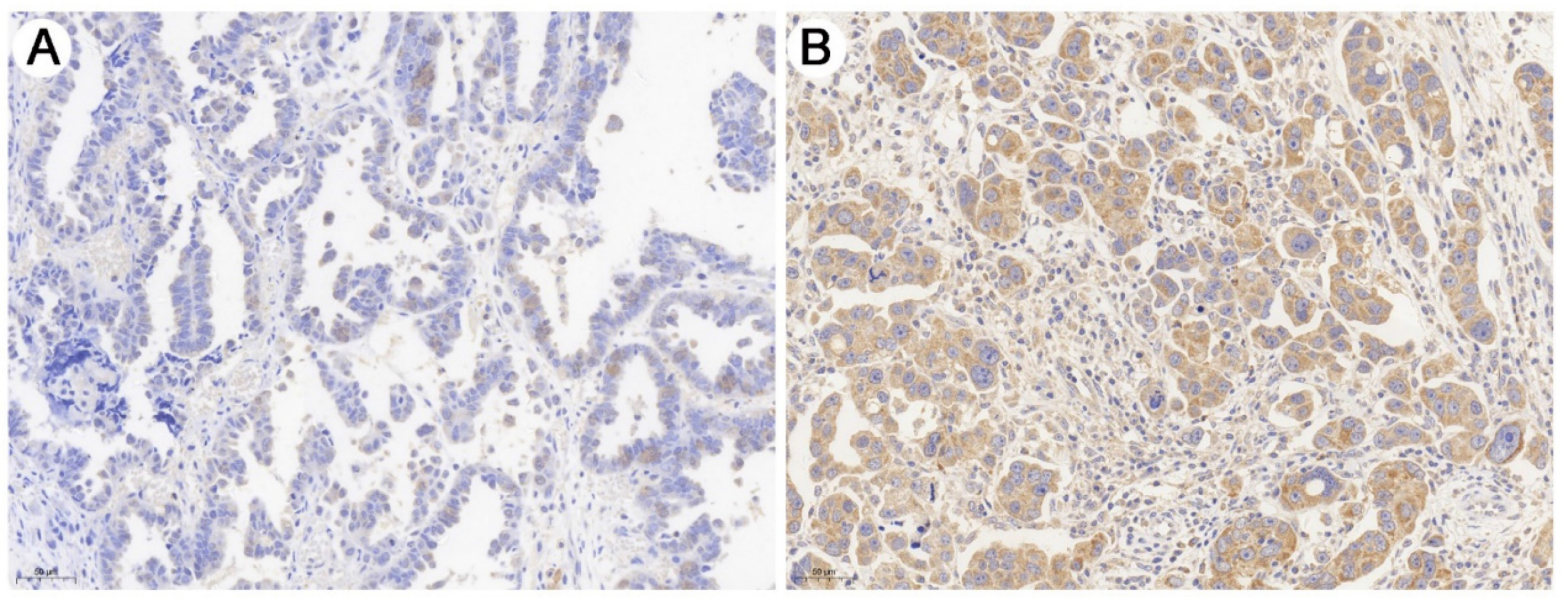
Representative image of immunohistochemical staining of the HGSOC sections with anti- DEPDC1B antibody. (A) HGSOC with low DEPDC1B expression. (B) HGSOC with high DEPDC1B expression.

As shown in Fig. 1, immunohistochemical staining showed that DEPDC1B was predominantly localized in the cytoplasm. Of the 60 SOC patients, 18 (30.0%) had high DEPDC1B expression and 42 (70.0%) had low DEPDC1B expression. DEPDC1B expression was associated with response to first line chemotherapy, and DEPDC1B expression was higher in platinum-resistant patients than in platinum-sensitive patients (*P* = 0.013). However, our results did not show any significant association between DEPDC1B expression and age, preoperative CA125 level, ascites status, location, FIGO stage, and residual disease (*P* > 0.05 for all; Table 1).

### High DEPDC1B expression predicts poor outcomes

**Figure 2:**
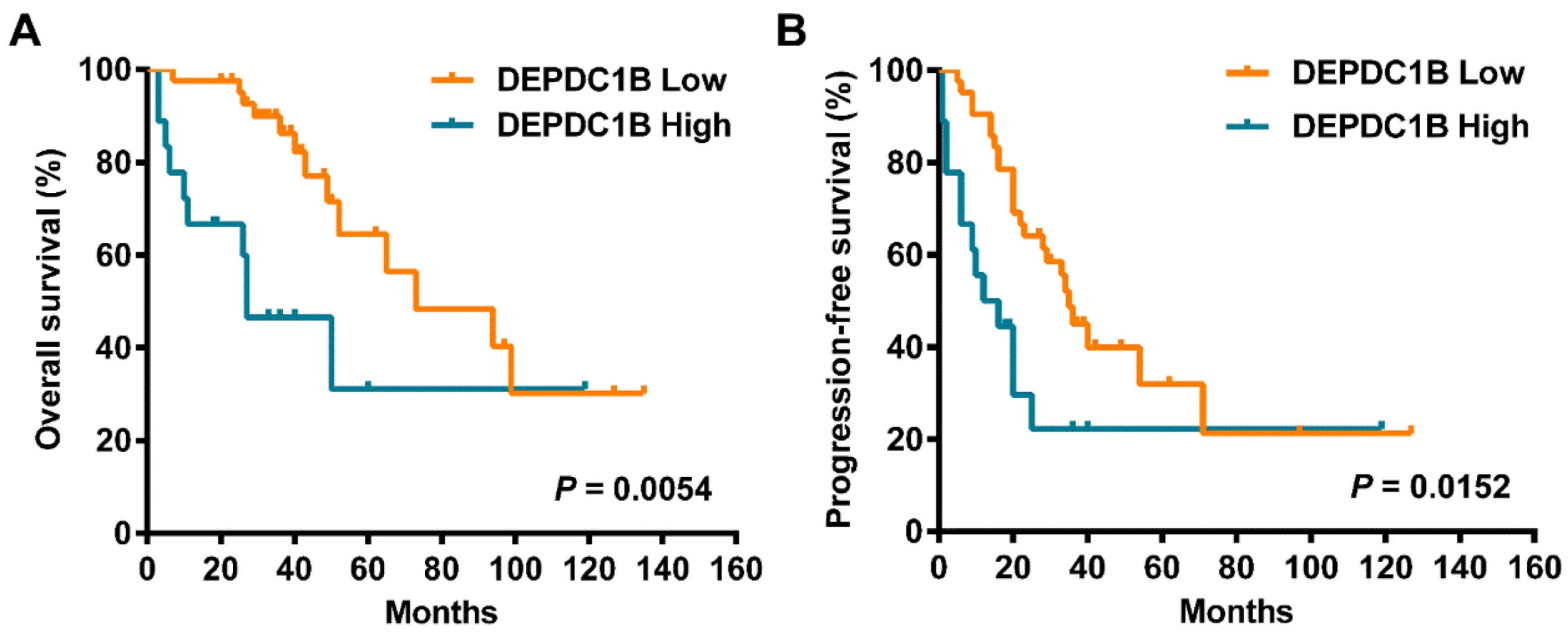
Kaplan‑Meier survival curves assessing (A) OS and (B) PFS times of patients with HGSOC according to DEPDC1B expression status.

Cox univariate analysis showed that increased DEPDC1B expression was correlated with reduced overall survival (OS) time (HR = 3.080, 95% CI = 1.337 to 7.098, *P* = 0.008; Table 2). Moreover, increased DEPDC1B expression was correlated with reduced progression-free survival (PFS) time (HR = 2.249, 95% CI = 1.136 to 4.455, *P* = 0.020; Table 2). Kaplan-Meier analysis further confirmed that patients with high tumor DEPDC1B expression had worse OS (*P* = 0.0054) and PFS (*P* = 0.0152) (Fig. 2). In addition, in univariate analysis, FIGO stage (*P* = 0.001) and response to first line chemotherapy (*P* < 0.001) showed a significant impact on OS. Similarly, in univariate analysis, FIGO stage (*P* < 0.001) and response to first line chemotherapy (*P* < 0.001) showed a significant impact on PFS. However, no significant associations were found between age, preoperative CA125 level, ascites status, location, residual disease and OS/PFS (*P* > 0.05 for all). Multivariate analysis demonstrated that FIGO stage (*P*= 0.024), response to first line chemotherapy (*P* = 0.021) and DEPDC1B expression (*P* = 0.023) independently predicted OS (Table 2). FIGO stage (*P* = 0.006) and response to first line chemotherapy (*P* < 0.001) independently predicted PFS. DEPDC1B expression was not an independent prognostic factor for PFS (*P* = 0.131, Table 2).

### Ectopic expression of DEPDC1B promotes the proliferation of EOC cells

**Figure 3:**
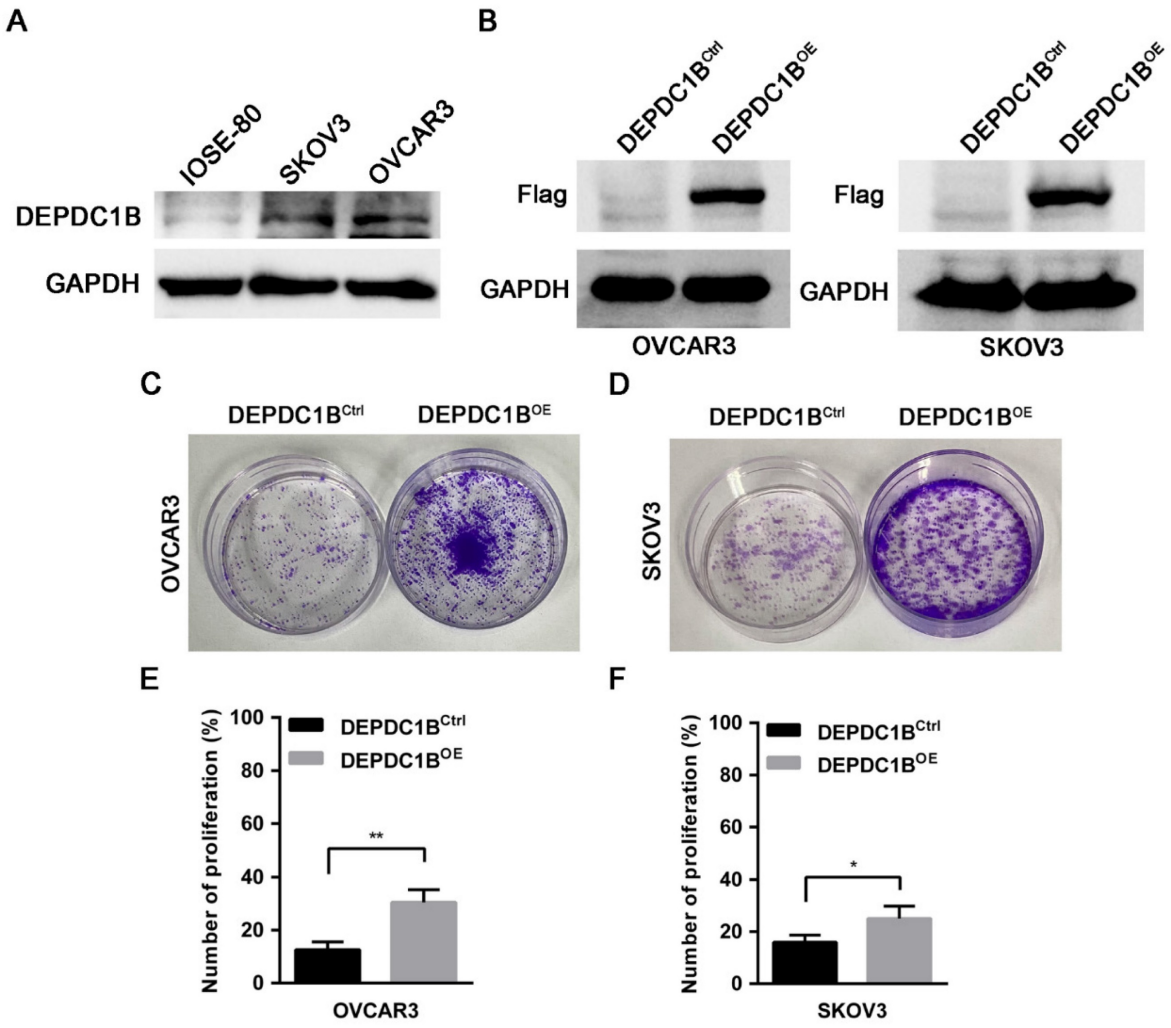
DEPDC1B promotes the proliferation of EOC cells. (A) Protein levels of DEPDC1B in human normal ovarian cell line IOSE-80, and the EOC cell lines OVCAR3 and SKOV3 were determined by western blot assays. (B) Efficiency of DEPDC1B upregulation was confirmed by western blot in stable DEPDC1B overexpression (DEPDC1B^OE^) and control (DEPDC1B^Ctrl^) OVCAR3 and SKOV3 cells. (C, D) Representative images from colony formation assay in DEPDC1B^OE^ and DEPDC1B^Ctrl^ OVCAR3 (C) and SKOV3 (D) cells. (E, F) EdU cell proliferation assay in DEPDC1B^OE^ and DEPDC1B^Ctrl^ OVCAR3 (E) and SKOV3 (F) cells. Data shown are the mean ± SEM of three independent experiments. ^*^, *P* < 0.05, ^**^, *P* < 0.01.

To determine the effects of DEPDC1B on the biological behaviors of EOC cells, we first examined the expression of DEPDC1B in the human normal ovarian cell line IOSE-80 and the EOC cell lines OVCAR3 and SKOV3. Western blot showed that OVCAR3 and SKOV3 cells expressed DEPDC1B at higher level as compared with IOSE-80 cells (Fig. 3A). Previous study reported that knockdown of DEPDC1B inhibited the proliferation of malignant melanoma cells 7. We next asked whether DEPDC1B could regulate the proliferation of EOC cells. OVCAR3 and SKOV3 cells stably expressing DEPDC1B (DEPDC1B^OE^) or control (DEPDC1B^Ctrl^) were generated by lentivirus infections and puromycin selection. The overexpression of DEPDC1B was confirmed by western blot analysis (Fig. 3B). Investigation of DEPDC1B regulation of EOC cell proliferation indicated that overexpression of DEPDC1B significantly increased the number of colonies compared with control cells (Fig. 3C, D). Subsequent EdU cell proliferation assays again confirmed the above findings in OVCAR3 and SKOV3 cells (Fig. 3E, F and Supplementary Fig. 1). To confirm the specificity of our findings, the effect of ectopic expression of DEPDC1B on A549 and BEL-7404 cell growth was investigated. Colony formation assay showed that ectopic expression of DEPDC1B did not significantly affect the number of colonies compared with control cells (Supplementary Fig. 2).

### DEPDC1B enhances AKT phosphorylation at Ser473 in EOC cells

**Figure 4:**
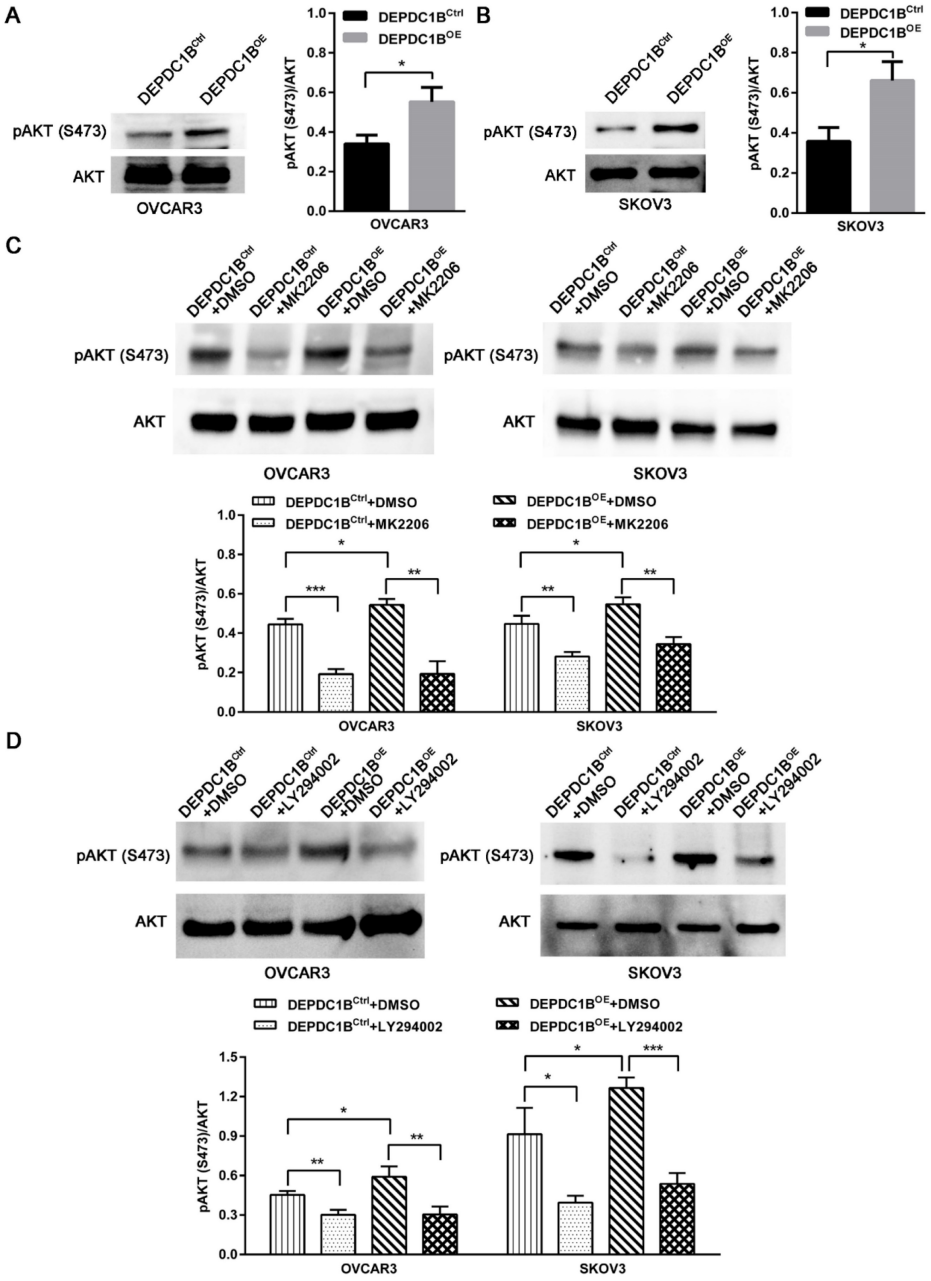
DEPDC1B enhances AKT phosphorylation at Ser473 in EOC cells. (A, B) AKT and pAKT (S473) expression were analyzed by western blot in DEPDC1B^OE^ and DEPDC1B^Ctrl^ OVCAR3 (A) and SKOV3 (B) cells. The western blot is shown at the left, and densitometry result is shown at the right. Data shown are the mean ± SEM of three independent experiments. ^*^, *P* < 0.05. (C) Western blot showing a reduction in pAkt at Ser473 on treatment with MK2206. The western blot is shown at the top, and densitometry result is shown at the bottom. Data shown are the mean ± SEM of three independent experiments. ^*^, *P* < 0.05, ^**^, *P* < 0.01, ^***^, *P* < 0.001. (D) Western blot showing a reduction in pAkt at Ser473 on treatment with LY294002. The western blot is shown at the top, and densitometry result is shown at the bottom. Data shown are the mean ± SEM of three independent experiments. ^*^, *P* < 0.05, ^**^, *P* < 0.01, ^***^, *P* < 0.001.

We next examined the effects of DEPDC1B up-regulation on pAKT expression. As shown in Fig. 4A and 4B, ectopic expression of DEPDC1B enhanced AKT phosphorylation at Ser473 in both OVCAR3 and SKOV3 cells. As expected, MK2206, an AKT inhibitor, suppressed the AKT phosphorylation at Ser473 that is induced by DEPDC1B up-regulation (Fig. 4C). Similar results were observed after treatment with PI3K inhibitor LY294002 in both OVCAR3 and SKOV3 cells (Fig. 4D). Taken together, these data suggest that ectopic expression of DEPDC1B could enhance AKT phosphorylation at Ser473.

### DEPDC1B promotes the proliferation of EOC cells by enhancing AKT phosphorylation

**Figure 5:**
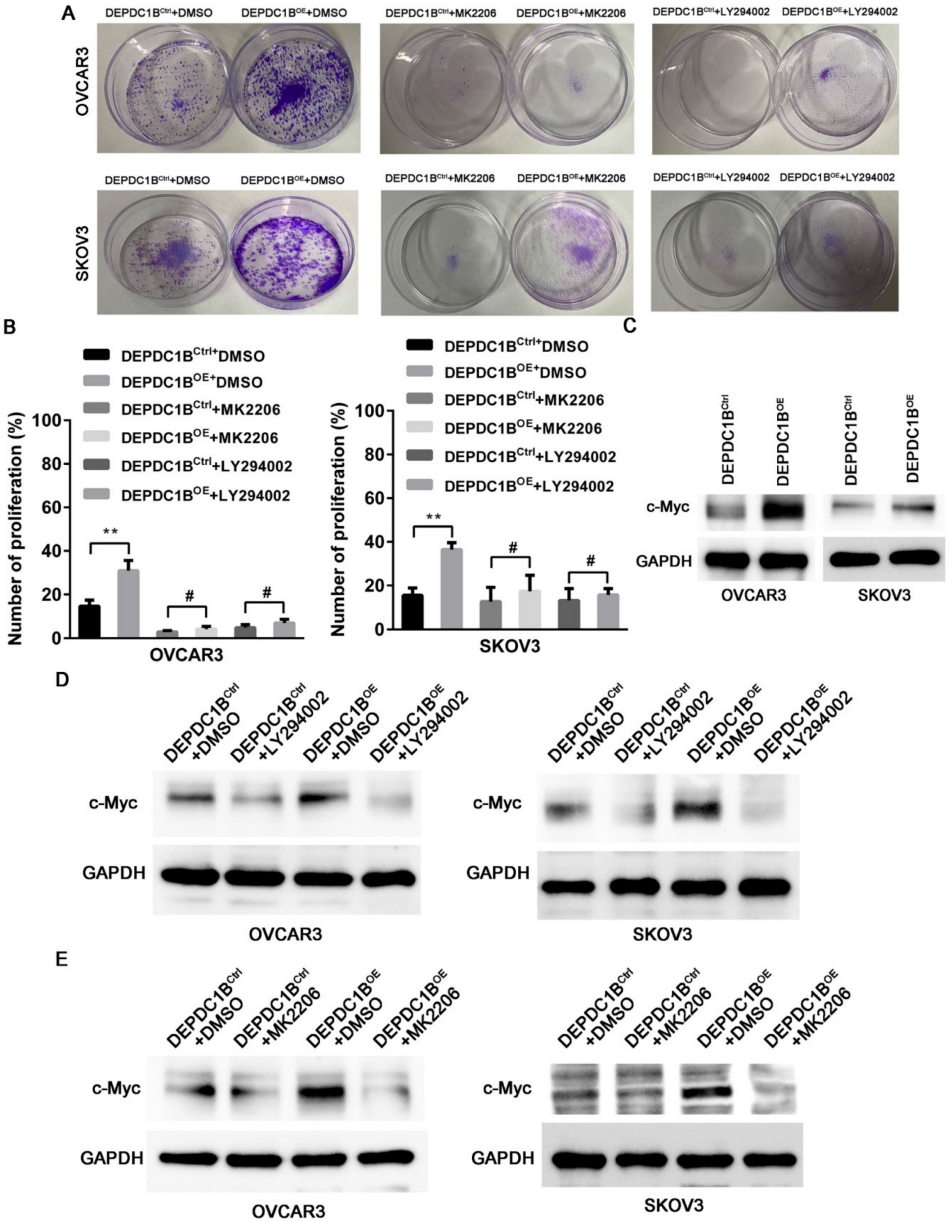
DEPDC1B promotes the proliferation of EOC cells by enhancing AKT phosphorylation. (A) Colony formation assay shows reduction in the number of colonies on treatment with MK-2206 and LY294002 in DEPDC1B^OE^ and DEPDC1B^Ctrl^ OVCAR3 and SKOV3 cells. (B) EdU cell proliferation assay shows reduction in cell proliferation on treatment with MK-2206 and LY294002 in DEPDC1B^OE^ and DEPDC1B^Ctrl^ OVCAR3 and SKOV3 cells. Data shown are the mean ± SEM of three independent experiments. ^**^, *P* < 0.01, ^#^, *P* > 0.05. (C) Protein levels of c-Myc in DEPDC1B^OE^ and DEPDC1B^Ctrl^ OVCAR3 and SKOV3 cells were determined by western blot assays. (D) Western blot showing a reduction in c-Myc expression on treatment with LY294002 in DEPDC1B^OE^ and DEPDC1B^Ctrl^ OVCAR3 and SKOV3 cells. (E) Western blot showing a reduction in c-Myc expression on treatment with MK2206 in DEPDC1B^OE^ and DEPDC1B^Ctrl^ OVCAR3 and SKOV3 cells.

We next examined the effects of MK2206 and LY294002 on DEPDC1B-mediated cell proliferation. As shown in Fig. 5A, treatment with MK2206 and LY294002 significantly suppressed the number of colonies that is induced by DEPDC1B up-regulation in both OVCAR3 and SKOV3 cells. Similar results were observed in OVCAR3 and SKOV3 cells using EdU cell proliferation assays (Fig. 5B and Supplementary Fig. 3 and 4). Previous study reported that Akt influences cell proliferation by increasing c-Myc expression 11. We next investigated the role of DEPDC1B in c-Myc expression. Western blot showed that ectopic expression of DEPDC1B increased c-Myc expression in both OVCAR3 and SKOV3 cells (Fig. 5C). Moreover, LY294002 suppressed c-Myc expression and decreased DEPDC1B-induced c-Myc expression (Fig. 5D). Similar results were observed after treatment with MK2206 in both OVCAR3 and SKOV3 cells (Fig. 5E). Taken together, these data suggest that ectopic expression of DEPDC1B promotes the proliferation of EOC cells via enhancing AKT phosphorylation at Ser473 and increasing c-Myc expression.

## Discussion

EOC is a “silent killer” and the majority of patients present with stage III/IV disease 2. Cytoreductive surgery, platinum-taxane chemotherapy have been remained the standard therapy of EOC for decades 12. Recently, EOC patients benefit from the development of targeted drugs 13. Despite these advances, the overall survival of EOC is still somber 14, 15.

Currently, DEPDC1B is reported to be overexpressed in several tumors. Bai et al. 9 reported that high expression level of DEPDC1B contributed to progression and affected the prognosis of prostate tumor patients. DEPDC1B acted as a tumor promoter for malignant melanoma and might be a novel therapeutic target for malignant melanoma 7. Our study showed that DEPDC1B expression was associated with response to first line chemotherapy, and it was higher in platinum-resistant patients than in platinum-sensitive patients. However, our results did not show any significant association between DEPDC1B expression and age, preoperative CA125 level, ascites status, location, FIGO stage, and residual disease. Moreover, our study demonstrated that increased DEPDC1B expression was correlated with reduced OS and PFS time in patients with HGSOC. Furthermore, multivariate analysis showed that DEPDC1B independently predicted OS in patients with HGSOC. However, DEPDC1B expression was not an independent prognostic factor for PFS in patients with HGSOC. Actually, research concerning the expression and mechanism of DEPDC1B in human cancers was still very limited and needs further investigation. Boudreau et al. 16 reported that DEPDC1B is a cell cycle-regulated molecule. High expression level of DEPDC1B in MDA-MB-231 human breast cancer cells was correlated with enhanced p-ERK expression, cell proliferation and cell survival. DEPDC1B could interact with Rac1, thereby regulating the proliferation, invasion, migration, and survival of tumor cells 5, 17. Yang et al. 8 demonstrated that DEPDC1B was increased in non-small cell lung cancer and could promote cell migration and invasion through activating the Wnt/β-catenin pathway. Our study demonstrated that DEPDC1B could promote the proliferation of OVCAR3 and SKOV3 cells by enhancing AKT phosphorylation at Ser473. Treatment with MK2206 and LY294002 significantly suppressed the proliferation that is induced by DEPDC1B up-regulation in both OVCAR3 and SKOV3 cells. We further elucidate the underlying mechanism associated with DEPDC1B up-regulation in EOC cell proliferation. We demonstrated that ectopic expression of DEPDC1B increased c-Myc expression in both OVCAR3 and SKOV3 cells. Moreover, MK2206 and LY294002 suppressed c-Myc expression and decreased DEPDC1B-induced c-Myc expression. Our findings indicate that ectopic expression of DEPDC1B promotes the proliferation of EOC cells via enhancing AKT phosphorylation at Ser473 and increasing c-Myc expression.

In conclusion, the present study provided the evidence for the first time that DEPDC1B expression was correlated with reduced OS and PFS time in patients with HGSOC. DEPDC1B independently predicted OS in patients with HGSOC. Our study also demonstrated that DEPDC1B could promote the proliferation of EOC cells via enhancing AKT phosphorylation at Ser473 and increasing c-Myc expression.

## Supplementary Material

Supplementary figures.

## Figures and Tables

**Table 1:** Relationship between expression levels of DEPDC1B and clinicopathological features of 60 HGSOC specimens

Variables	*n*	DEPDC1B		*P* value
		Low	High	
Age (years)				0.366
≤55	32	24	8	
>55	28	18	10	
Preoperative CA125 level (U/mL)				0.576
≤35	4	2	2	
>35	56	40	16	
Ascites (mL)				0.303
≤500	41	27	14	
>500	19	15	4	
Location				0.815
Unilateral	22	15	7	
Bilateral	38	27	11	
FIGO stage				0.232
I / II	20	16	4	
III / IV	40	26	14	
Residual disease				0.346
R0	16	13	3	
non-R0	44	29	15	
Response to first line chemotherapy				0.013
Platinum-sensitive	47	37	10	
Platinum-resistant	13	5	8	

R0: No residual tumor

**Table 2:** Univariate and multivariate analysis of prognostic factors in 60 HGSOC specimens

	Univariate analysis		Multivariate analysis	
	HR (95% CI)	*P*	HR (95% CI)	*P*
OS				
Age	1.919 (0.827 - 4.452)	0.129		
Preoperative CA125 level	2.176 (0.290 - 16.322)	0.450		
Ascites	1.115 (0.456 - 2.726)	0.812		
Location	1.635 (0.680 - 3.929)	0.272		
FIGO stage	8.188 (2.292 - 29.252)	0.001	4.809 (1.226 - 18.869)	0.024
Residual disease	2.260 (0.758 - 6.736)	0.143		
Response to first line chemotherapy	6.037 (2.573 - 14.163)	<0.001	3.061 (1.182 - 7.932)	0.021
DEPDC1B expression	3.080 (1.337 - 7.098)	0.008	2.778 (1.148 - 6.725)	0.023
PFS				
Age	1.449 (0.760 - 2.765)	0.260		
Preoperative CA125 level	0.541 (0.165 - 1.775)	0.311		
Ascites	1.256 (0.638 - 2.472)	0.510		
Location	1.365 (0.686 - 2.713)	0.375		
FIGO stage	6.707 (2.325 - 19.354)	<0.001	4.712 (1.549 - 14.332)	0.006
Residual disease	2.063 (0.902 - 4.717)	0.086		
Response to first line chemotherapy	60.355 (15.498 - 235.046)	<0.001	39.563 (9.750 - 160.539)	<0.001
DEPDC1B expression	2.249 (1.136 - 4.455)	0.020	1.760 (0.845 - 3.669)	0.131
