# Supplementary material for: Role of DEP domain-containing protein 1B (DEPDC1B) in epithelial ovarian cancer
Source: J Cancer. 2023 Mar 27;14(5):784–92. doi: 10.7150/jca.78423 (PMC10088892; doi:10.7150/jca.78423)
Supplement: Supplementary file 1 — Supplementary figures. [file jcav14p0784s1.pdf]

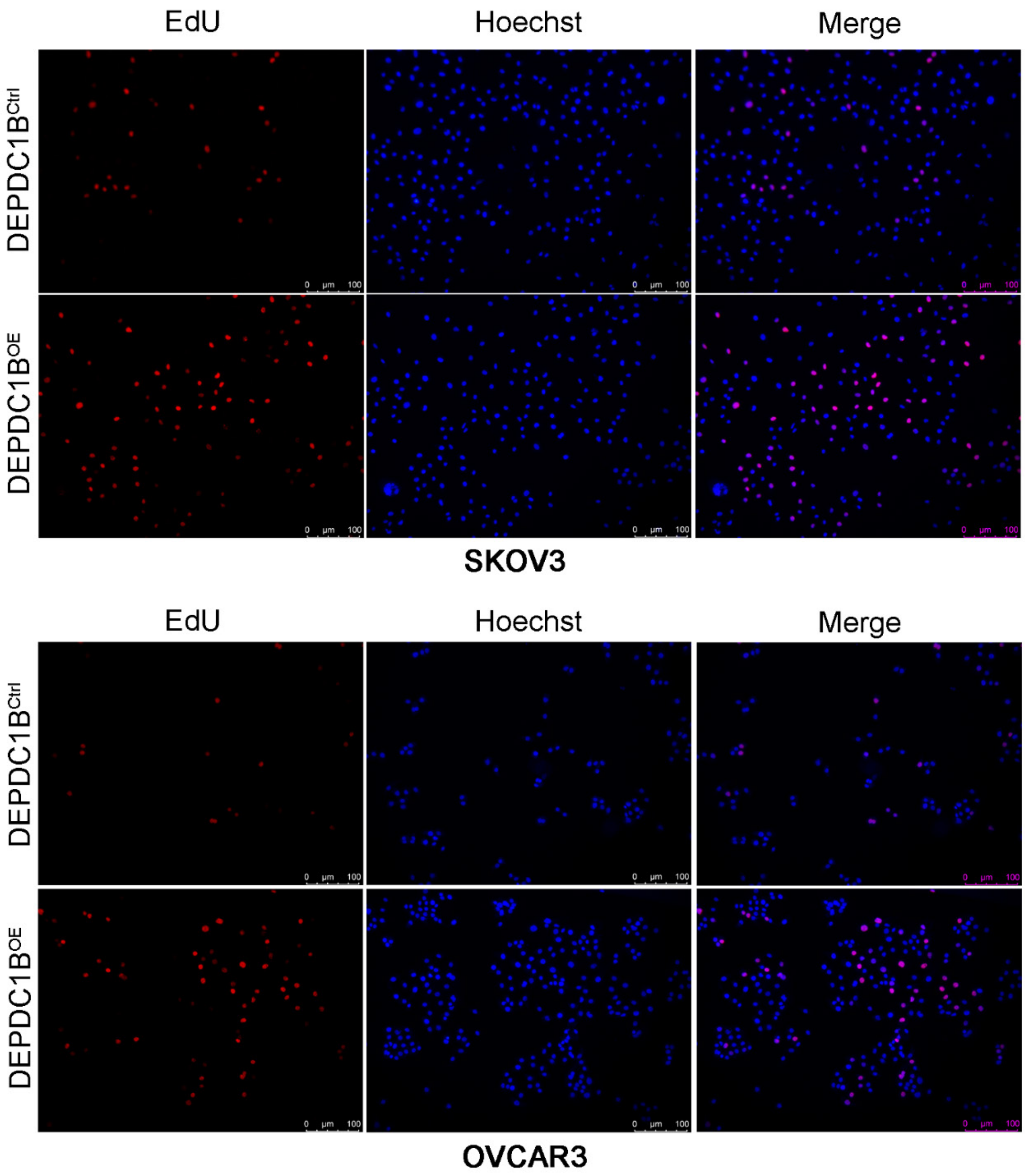

Supplementary Figure 1 Representative images from EdU cell proliferation assay in DEPDC1B^{OE} and DEPDC1B^{Ctrl} SKOV3 and OVCAR3 cells.

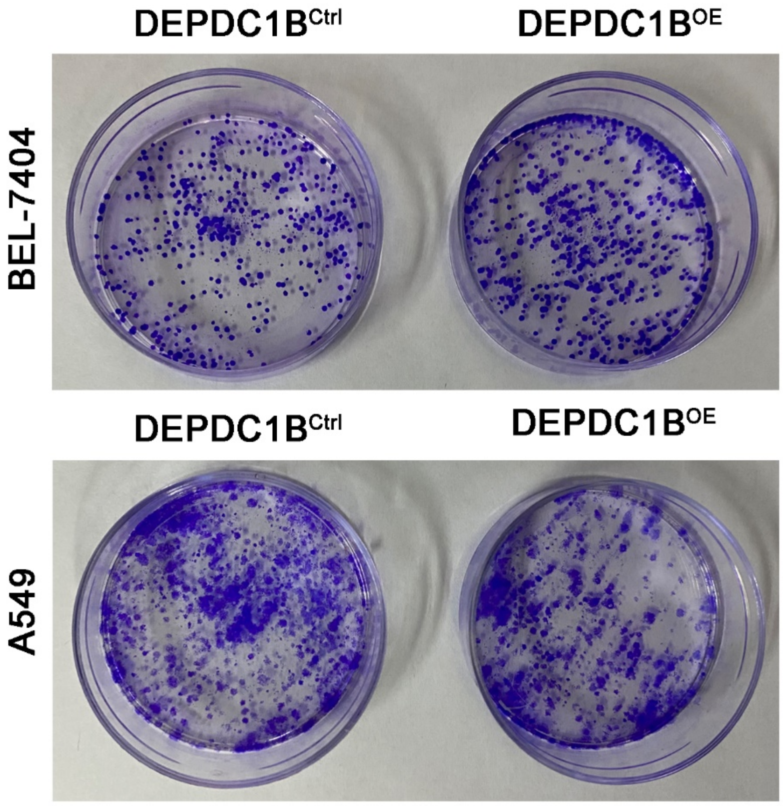

Supplementary Figure 2 Representative images from colony formation assay in DEPDC1B^{OE} and DEPDC1B^{Ctrl} BEL-7404 and A549 cells.

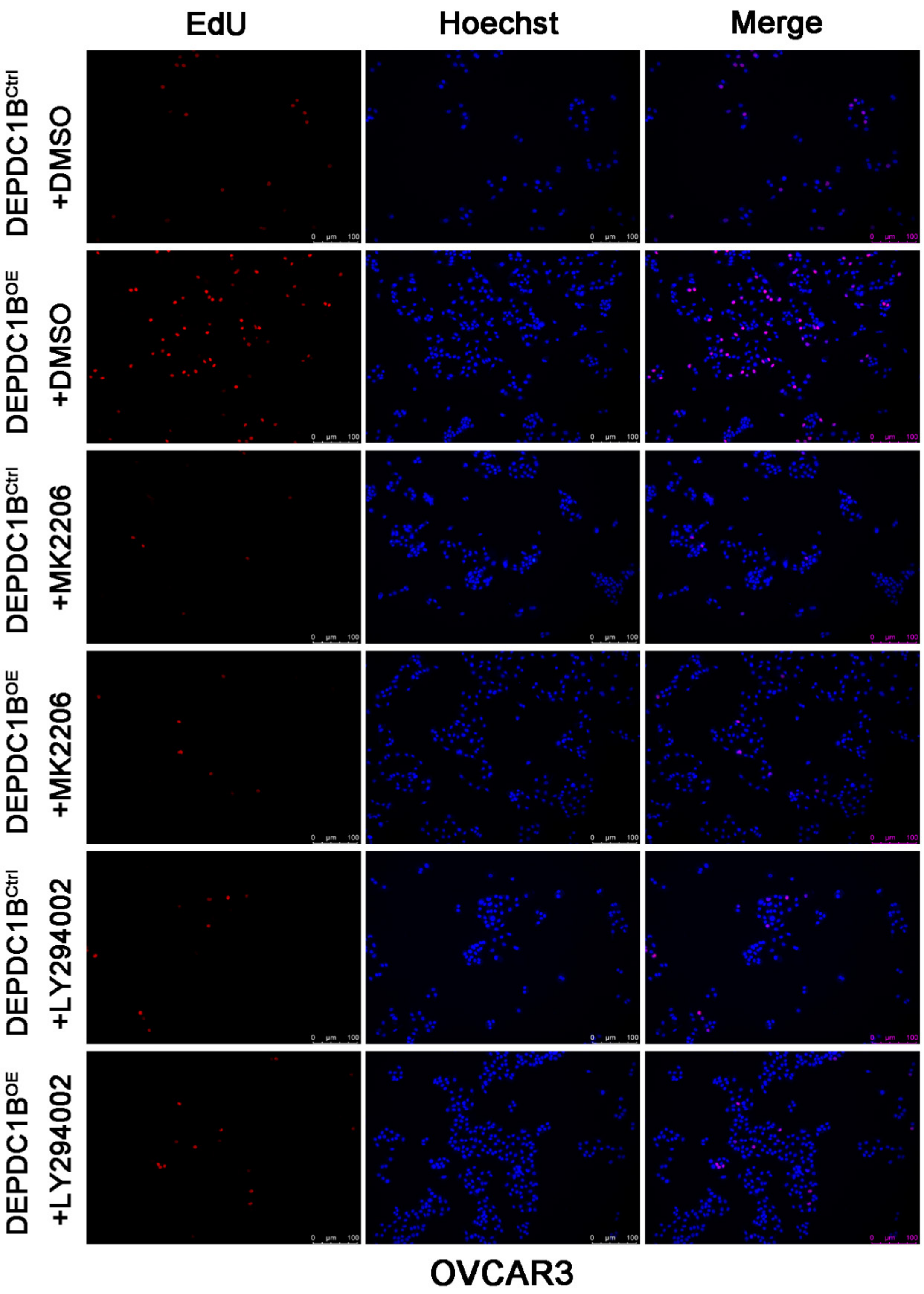

Supplementary Figure 3 Representative images from EdU cell proliferation assay in DEPDC1B^{OE} and DEPDC1B^{Ctrl} OVCAR3 cells treated with MK-2206 or LY294002.

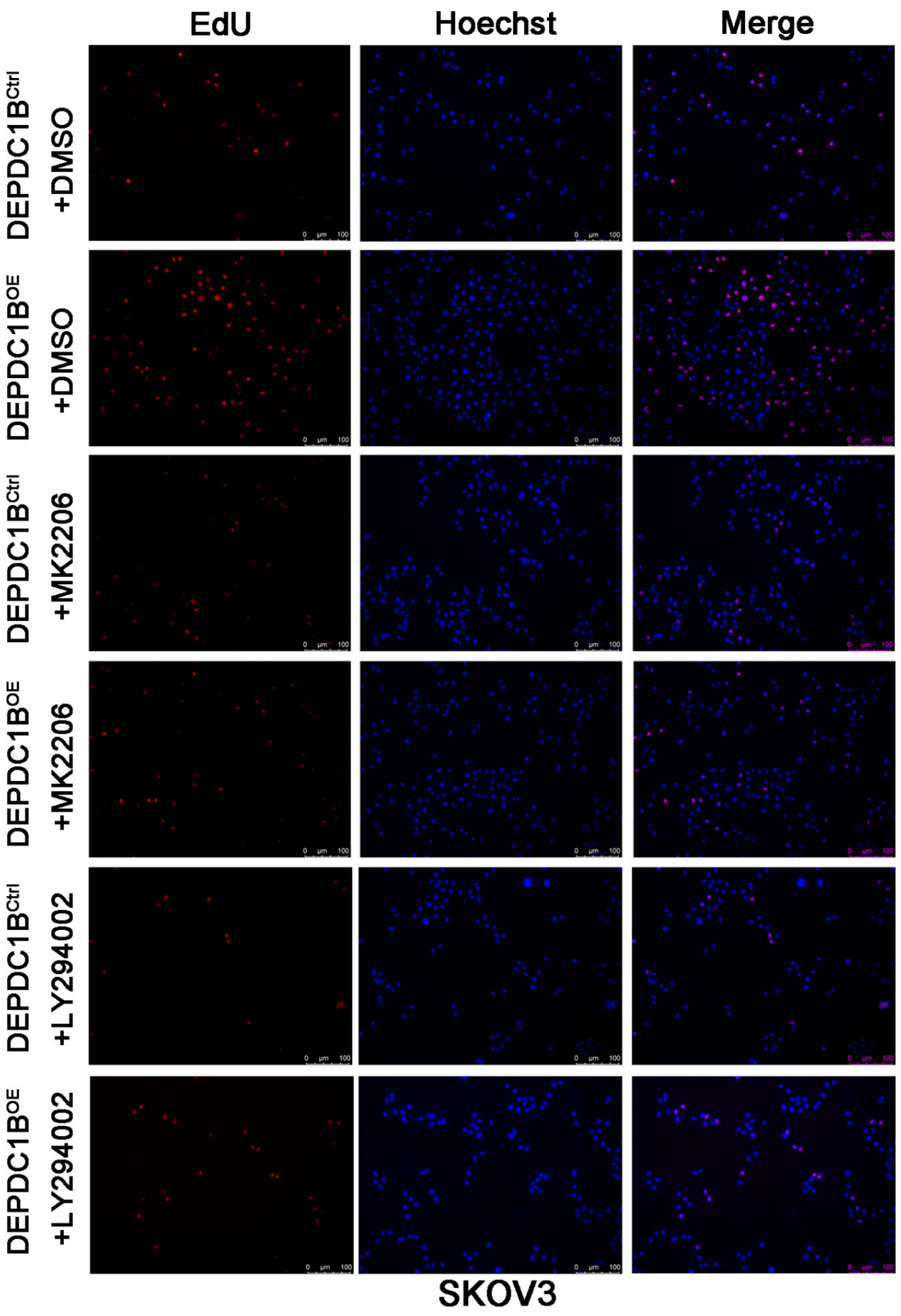

Supplementary Figure 4 Representative images from EdU cell proliferation assay in DEPDC1B^{OE} and DEPDC1B^{Ctrl} SKOV3 cells treated with MK-2206 or LY294002.
